# Depression in the elderly in Karachi, Pakistan: a cross sectional study

**DOI:** 10.1186/1471-244X-13-181

**Published:** 2013-07-03

**Authors:** Mehreen Anwar Bhamani, Mehtab S Karim, Murad Moosa Khan

**Affiliations:** 1 Department of Community Health Sciences, Aga Khan University, Stadium Road, P.O. Box 3500, Karachi, 74800, Pakistan; 2 Department of Psychiatry, Aga Khan University, Karachi, Pakistan

**Keywords:** Psychiatric problems, Depression, Elderly, Aging, Geriatric, Prevalence

## Abstract

**Background:**

Depression in elderly is a major global public health concern. There has been no population-based study of depression in the elderly in Pakistan. The aim of the study was to estimate the prevalence of depression and its association with family support of elderly (age 60 years and above) in Karachi, Pakistan.

**Methods:**

A population based cross-sectional study was carried out in Karachi from July-September 2008. Questionnaire based interviews were conducted with individuals (n = 953) recruited through multi-stage cluster sampling technique, using the 15- item Geriatric Depression Scale (GDS).

**Results:**

Prevalence of depression was found to be 40.6%, with a higher preponderance in women than men (50% vs. 32%). Elderly currently not living with their spouses were 60% more depressed than those living with their spouses (Adjusted OR = 1.6, 95% CI = 1.3-2.1). Elderly who did not consider their children as future support were twice as likely to be depressed as those considering their children to be old age security (Adjusted OR = 2.1, 95% CI = 1.4-3.1). An increase in one male adult child showed 10% decrease in depression after adjusting for other variables (Adjusted OR = 0.9, 95% CI = 0.8-0.9).

**Conclusion:**

A relatively high prevalence of depression was found in the elderly in Karachi. There appeared to be a strong association between depression and family support variables such as living with spouse, considering children as future security and number of male adult children in the sample studied. Mental wellbeing of the elderly in Pakistan needs to be given consideration in the health policy of the country. In collectivistic societies like Pakistan family support plays an important part in mental health of the elderly that needs to be recognized and supported through various governmental and non-governmental initiatives.

**Keypoints:**

Assessment of depression in elderly, Cross-sectional study in Karachi-Pakistan

## Background

Globally, evolving knowledge and practices coupled with socio-economic development has led to increase in average life expectancy on the one hand and a substantial decline in fertility rates on the other. These changes have given rise to population aging, defined as a shift in the average age of population from younger to older [1,2]. An aging population leads to increased burden of chronic diseases, physical disabilities, mental illnesses and other co-morbidities [3]. Health care systems throughout the world face major challenges to improve the overall health and quality of life of their elderly population [4,5]. Assessing the needs and problems of the elderly therefore becomes an important first step in the process of addressing this. This task is more challenging for lower and middle income (LAMI) countries where population aging has been much faster [6-8] but resources are severely constrained. United Nation 2008 estimates suggest that the growth rate of the elderly population in developing countries (3%) is approximately twice that of developed countries (1.9%) [9].

Pakistan is a South Asian Islamic developing country with a current estimated population of 180 million [1]. It has a dependency ratio of 0.75 [10] and is already facing a challenge of chronic disease burden that are projected to account for 42% of all deaths [11]. WHO estimates that the health workforce in Pakistan of 8.1 physicians and 5.9 nurses/midwives per 10,000 population is quite low compared to the regional average of 10.9 and 15.6 per 10,000 population respectively [12]. The country is therefore, faced with a major deficit in healthcare resources to meet the healthcare demands of the growing population. In this context the elderly population (60+ years) in Pakistan, estimated to increase from 6.1% in 2009 to 14.9% by 2050 [9] highlights the inadequacy of the country’s health system to deal with the increased burden of diseases of the aging population [13,14]. Hence, it has become important to identify the magnitude of problems of the elderly to inform policy to cater to the health problems (both physical and mental) in this neglected but important group of population.

Depression, a major global public health problem is common in the elderly [15-20]. In both high income and LAMI countries, depression in elderly carries a huge burden, contributing to approximately one- sixth of all disability adjusted life years (DALYs) [21]. There has been no population-based study of depression in the elderly in Pakistan. Two previous health centre-based studies on elderly patients reported prevalence of depression as 20% -23% [22,23].

Of the various risk factors, family support has a strong association with depression in the elderly.

Families play a prominent role in the life of an elderly especially in Asian culture as most Asian countries have a joint family system [24]. Although rapidly evolving urbanization has affected the Asian family structure, in countries like Pakistan, the family continues to be a significant source of security and support to the elderly [25,26]. The elderly hold an authoritative place in a family and their position demands great respect and admiration [24]. Besides relying on their spouse, the elderly also seek support from their children especially male children as females usually leave their parent’s place after getting married [24]. This includes social, financial, physical as well as emotional support and has been reported by several studies to be associated with their mental health [27,28].

Based on these observations, the objectives of our study was to determine the prevalence of depression and its association with family support in elderly population (age 60 and above) in Karachi, Pakistan.

## Methods

### Study design and setting

This cross-sectional study was conducted from July to September 2008 in Karachi, Pakistan’s largest city and its main economic and cultural hub. Karachi’s current population (estimated on population census of 1998) is approximately 15 million [29]. For administrative purposes, the city is divided into 18 towns. Karachi has a culturally and ethnically diverse population as people from different parts of the country come here to seek employment.

### Study participants

Persons 60 years and above of both genders from study areas of Karachi, who gave consent to participate were included in the study. Our exclusion criteria included elderly people suffering from mental retardation, those with cognitive impairment and those with serious co-morbid medical conditions like cancer, advanced heart failure or terminal renal failure etc. For screening purpose, the Mini Mental State Examination (MMSE) was performed to assess cognitive impairment [30]. The purpose of performing cognitive function assessment was that the Geriatric Depression Scale (GDS) is not valid for cognitively impaired individuals [31]. Those scoring less than 23 (for literate) or 21 (for illiterate) were labeled to have cognitive impairment and therefore, were excluded from the study [32-34]. Participants with serious co-morbid conditions were excluded. This was based on their current medical reports, physical conditions and their response to specific questions about their ability to interact with an interviewer for more than half an hour.

### Study sample and recruitment

The sample size was calculated using Epi info version 6. We took prevalence of depression as 20% (based on a previous health center based study of elderly depression in Karachi) [22] and assumed the proportion of elderly not having family support as 50%. Taking into account 95% confidence intervals, 80% power and odd ratio of 2:00 (for design effect using a multistage cluster sampling) [35] and an anticipated non-response rate, we obtained the final sample size of approximately 950.

The sample was drawn with the help of a population-based representative sample survey using multi-stage cluster sampling technique. The sampling frame was based on the data generated from the most recent population census as updated by Federal Bureau of Statistics (FBS). FBS has divided the city of Karachi into 4000 blocks. Each block comprises of minimum 250 to maximum of 350 households.

The number of blocks to be sampled was determined based on the estimation of the minimum number of households having elderly people in each block. We sampled 46 blocks for our study from the complete list of the blocks through simple random sampling. Each block in the list was assigned a number. By generating random numbers in computer we drew 46 blocks on random basis. From each sampled block, 21 households were selected with equal probability, through systematic sampling, in order to achieve the calculated sample size of 950. Systematic sampling involves a random selection of a house and sampling every K^th^ house (where K represents the constant sampling interval based on the number of households to be sampled from total households in each block). Only one elderly person from each household was enrolled in the study. In the presence of two or more eligible elderly persons in a household, balloting was done to select one of them.

Once identified, the study subject was screened for eligibility criteria. Those who met the criteria and agreed to participate were introduced to the study, its objectives, procedures and possible consequences. All study subjects were interviewed by trained interviewers.

The data collection team consisted of six interviewers and two field supervisors, all of whom were fluent in Urdu and were well-trained for the purpose. Confidentiality was assured at the time of data collection and random spot checks were performed by the principal investigator to ensure data quality.

### Questionnaire

A detailed questionnaire was used as a data collection tool. The questionnaire was developed in English language, translated into Urdu and back translated in English by experts proficient in both languages, and checked for consistency. Since Urdu is the most widely spoken and understood language in Karachi, we used the questionnaire in Urdu. A manual of operation was prepared for the interviewers to ensure uniformity in the data collection process. The questionnaire was pre-tested on 50 elderly individuals (5% of the sample size) to assess the flow and clarity of questions. Changes were made accordingly in the final questionnaire.

The final questionnaire included information regarding socio-demographic characteristics, functional status and physical activity levels, family support profile, and depression status of study participants. Refer additional questionnaire file for more details [see Additional file 1].

### Study variables

“Depression Status” was considered as a dependent variable and was included in the analysis as binary variable [yes, no]. The variable was assessed with the help of 15-item version of Geriatric Depression Scale (GDS) [36,37]. This is the most widely used scale to assess depression in elderly both for clinical and research purposes [31]. The scale has not been validated in our elderly population however, it has been used in two previous health centre-based studies on elderly in Karachi [22,23]. GDS-15 has been validated in many different languages though not in Urdu. The scale was translated in Urdu, back translated into English and checked for consistency with the help of subject experts. It was pre-tested in 50 subjects (about 5% of the sample) and changes made accordingly. Elderly, scoring 5 and above on GDS were considered to have depression, as this cutoff is reported to have high sensitivity and specificity in previous studies [22,23].

Socio-demographic information of elderly included age, gender [men, women], employment status [working, not working], level of education [illiterate, informal education, primary to matriculation, above matriculation], ethnicity [Urdu speaking, Punjabi, Pashto, Sindhi/Balochi] and place of birth [India, within Pakistan but outside Karachi or within Karachi].

Functional status (ability to perform activities of daily living) was assessed using the 15-item Activities of Daily Living (ADL) scale [38,39]. The ADL scale has 10 questions related to non-instrumental and 5 questions related to instrumental activities of daily living (IADL). The ADL has been used in several studies to assess disability among elderly population [38,39]. The ADL involves questions related to self-care and daily routine including dressing, bathing, eating, toileting etc. The IADL includes things that people do to maintain their own independent lifestyle e.g.: shopping, housekeeping, using telephone etc. IADL are considered to be more difficult than ADLs because they require both physical and cognitive abilities [38,39]. The response to all 15 items was divided into “yes” and “no”. Each “yes” response was given score one out of fifteen. Since the scale has not been validated in our setting, no particular cutoffs are available to differentiate between disabled and non-disabled individuals. Hence, the variable is taken in a continuous form based on its scale analysis.

For assessing the level of physical activity, the amount of time spent by the subject in walking, lifting weights (up to 5 kg), meeting friends, purchasing items or other leisure activities was inquired. The larger the amount of time spent in such activities the higher the level of activity. The variable was divided into categories of “0-120 min/week”, “>120-310 min/week” and “>310 min/week” based on its scale analysis.

Family support variables included, marital status [living with spouse, not living with spouse], family structure [alone, nuclear, extended with children/relative], number of adult male and female children alive, and perception of the elderly about children as their future security [yes, no] [40,41], defined in terms of both economic and social support.

### Ethical considerations

Ethical approval was obtained from Ethical Review Committee, Aga Khan University (ERC-AKU). Written informed consent was obtained from each study participant. Participants who scored above the cut-off value and had a high probability of suffering from depression were offered free consultation with a psychiatrist at the department of psychiatry, AKU.

### Statistical analysis

Data was entered in Epi data version 3.1 and was analyzed using Statistical Analysis System (SAS), version 9.1. Since multi-stage cluster sampling technique was applied in which each cluster (block) was of different size, weighted analysis was performed by giving weights to each cluster (block) in proportion to its size [42,43]. The weights were allocated to each cluster (block) using the below formula:

W=NM/nm

Where “N” denotes the total number of clusters/blocks in Karachi.

“n” denotes the number of clusters/blocks sampled for study.

“M” denotes the total number of household in each sampled cluster/block.

“m” denotes the number of sampled households from each sampled cluster/block.

#### Data analysis was carried out in two steps

##### Descriptive statistics

Descriptive statistics were used to show the prevalence of depression in elderly. Weighted means and standard deviations for continuous variables and weighted frequencies/percentages for all categorical variables were estimated.

##### Univariate and multivariable analysis

Based on scoring of GDS, the dependent variable of “depression status” was created as a binary variable having categories ‘Yes’ and ‘No’. Data was analyzed using Binary Logistic Regression.

Univariate analysis was performed to study the effect of family support profile and other socio-demographic variables on the depression status. Assessment of family support was based on four variables: marital status, family structure, number of living adult male children and consideration of children as future security.

In addition, all other co-variates were regressed separately with the depression status in univariate analysis to get the crude estimates of their ORs and CIs. Among these covariates, level of education, self perception of health status, total time spent per week in physical activity were categorical variables whereas, score of ADL and IADL was included as a continuous variable in the analysis.

Multivariable analysis was carried out to study the independent association of family support with depression. Co-variates that showed p-value of < 0.25 at univariate level were included in the multivariable analysis for adjustment.

To develop a final multivariable model, we entered the variables in the model one by one. The final model included variables that were significant at p < 0.05. Tables were created for comprehensive viewing of results.

## Results

A total of 1023 subjects who fulfilled our inclusion criteria were contacted, of which 966 agreed to participate in the study. 13 individuals were found to be cognitively impaired on the MMSE and hence, excluded from the study. Thus, a total of 953 subjects were recruited and interviewed.

Prevalence of depression was found to be 40.6%, with a higher preponderance in women (50%) as compared to men (32%). All means and standard deviations for continuous variables and frequencies for categorical variables illustrated in the tables are weighted.

Table 1 shows the socio-demographic characteristics of participants. 53% were men. Majority were Muslims and Urdu speaking. Slightly over one-fourth of men were employed as compared to one-twelfth of women. There were more women (68%) compared to men (44%) with no formal education. 55% of the participants born outside Karachi had immigrated to Karachi from different parts of India (Pakistan came into being in August 1947 with the end of British rule and division of India).

**Table 1:** Socio-demographical profile of the elderly in Karachi

	**Men**	**Women**
	**n = 506**	**n = 447**
	**N (weighted%)**	**N (weighted%)**
**Age (weighted mean ± SD)**	67.8 (0.4)	66.2 (0.3)
**Religion**		
Muslims	452 (89.3)	401 (89.7)
Non-Muslims	54 (10.7)	46 (10.3)
**Employment status**		
Not Working	371 (73.3)	411 (91.9)
Working	135 (26.7)	36 (8.1)
**Marital status**		
Living with spouse	327 (64.6)	151 (33.8)
Not Living with spouse	179 (35.4)	296 (66.2)
**Level of education**		
Illiterate	220 (43.5)	304 (68.0)
Informal Education	44 (8.7)	55 (12.3)
Primary to Matriculation	177 (35.0)	75 (16.8)
Above Matriculation	65 (12.8)	13 (2.9)
**Ethnicity**		
Urdu Speaking	327 (64.6)	280 (62.6)
Punjabi/ Saraiki/Rajhistani	87 (17.2)	73 (16.3)
Pashto/Hindko	58 (11.5)	37 (8.3)
Sindhi/Balochi	34 (6.7)	57 (12.8)
**Place of birth**		
India	219 (43.3)	179 (40.0)
Within Pakistan other than	174 (34.4)	150 (33.6)
Karachi		
Karachi	113 (22.3)	118 (26.4)

Table 2 indicates the social support available from immediate family members. More elderly men as compared to women were living with spouse (64.6% vs 33.8%). Moreover, most study participants had good family support with 92% males and 94% females living with either their children or other relatives. A small percentage was living alone. On average, our study participants had close to 5 living children and most of them were adults. Around 80% of elderly persons considered children as their future security.

**Table 2:** Social support available to the elderly in Karachi from immediate family members

	**Men**	**Women**
	**n = 506**	**n = 447**
	**N (weighted%)**	**N (weighted%)**
**Ever married**		
Yes	493 (97.4)	442 (98.9)
No	13 (2.6)	5 (1.1)
**Marital status**		
Living with spouse	327 (64.6)	151 (33.8)
Not living with spouse	179 (35.4)	296 (66.2)
**Number of children born alive (weighted mean±SD)**	6.1 (0.3)	6.4 (0.3)
**Number of children currently alive (weighted mean±SD)**	5.5 (0.3)	5.9 (0.3)
**Number of only adult children alive (weighted mean±SD)**	4.6 (0.1)	5.1 (0.1)
**Number of adult male children alive (weighted mean±SD)**	2.4 (0.1)	2.8 (0.1)
**Number of adult female children alive (weighted mean±SD)**	2.2 (0.1)	2.4 (0.1)
**Number of family members living with elderly (weighted mean±SD)**	6.4 (0.2)	5.9 (0.1)
**Number of adult children living with elderly (weighted mean±SD)**	2.3 (0.1)	2.1 (0.1)
**Family structure**		
Alone	12 (2.4)	14 (3.1)
Nuclear	30 (5.9)	12 (2.7)
Extended with children	448 (88.5)	407 (91.1)
Extended with other	16 (3.2)	14 (3.1)
relatives		
**Consider children as future security**		
Yes	403 (79.6)	359 (80.3)
No	87 (17.2)	75 (16.8)
Don’t know	16 (3.2)	13 (2.9)

Table 3 indicates the association of family support with depression on univariate and multivariable analysis. Those subjects not currently living with their spouses were 60% more likely to be depressed than those living with their spouses (Adjusted OR = 1.6, 95% CI = 1.3-2.1). Moreover, the increasing number of male children showed a protective association with depression. Thus, an increase in one male child showed 10% protective odds against depression after adjusting for other variables (Adjusted OR = 0.9, 95% CI = 0.8-0.9). Elderly not considering children as their future support were twice likely to be depressed than those considering their children to be old age security (Adjusted OR = 2.1, 95% CI = 1.4-3.1).

**Table 3:** Association of family support with depression in Elderly of Karachi on Univariate and multivariable analysis

	**Depressed**	**Not Depressed**	**Unadjusted ORs**	**95% Confidence intervals**	**P-Value**
	**n = 387 N (weighted)**	**n = 566 N (weighted)**			
**Marital status**					
Living With Spouse	142	336	1.0		
Living Without Spouse	245	230	2.5	2.0-3.1	<0.01*
**Consider children as future security**					
Yes	296	466	1.0		
No	82	80	1.6	1.1-2.4	
Don’t Know	09	20	0.7	0.3-1.5	0.01*
**Family structure**					
Extended With Children	346	509	1.0		
Extended With Other Relatives	16	14	1.6	0.8-3.5	
Nuclear	15	27	0.8	0.4-1.5	0.49
Alone	10	16	0.9	0.5-1.8	
**Number of adult male children alive (weighted means+SD)**	2.5 (1.7)	2.6 (1.8)	0.9	0.8-1.0	0.17*
			**Adjusted ORs**	**95% Confidence intervals**	**P-Value**
**Marital status**					
Living With Spouse			1.0		
Living Without Spouse			1.6	1.3-2.1	<0.00**
**Consider children as future security**					
Yes			1.0		
No			2.1	1.4-3.1	<0.00**
Don’t Know			0.9	0.4-2.5	
**Number of adult male children alive**			0.9	0.8-0.9	0.01**
(as continuous)					

*P value <0.25

**P value <0.05

Adjusted for Level of Education, Self Perception of Health Status, Functional Status and Physical Activity Level.

The variables adjusted in the final multivariable model were level of education, self perception of health status, functional status and physical activity level.

## Discussion

To our knowledge, this is the first study that has estimated the prevalence of depression in the elderly population using a community based sample in Pakistan.

Our results show that approximately 40% of elderly population living in Karachi is suffering from depression, with higher rates in women than men. Elderly not living with spouse and not considering their children as future security were more likely to be depressed. Conversely, elderly having male children showed protection against depression. Although the cross-sectional design of the study could not establish the temporal relationship between family support and depression, the findings of our study provide important insights into the connection of family support with the mental health of elderly.

Given the fact that we used a population-based approach on a representative sample, our findings could be generalized to the elderly population of Karachi, though not to the country as a whole.

The household survey design made it impractical to access homeless elderly, those living at old peoples’ homes (there are a small number in Karachi) and severely depressed elderly admitted in hospitals. Similarly, the exclusion of those elderly who were cognitively impaired and those with severe medical co-morbidities might have underestimated the observed prevalence. In addition, there may have been bias associated with self-reporting and use of non-validated scales to assess depression and functional status.

The prevalence estimated by our study is approximately twice that of two previous studies on elderly attending a tertiary healthcare facility in Karachi, using the same scales and cutoff points [22,23]. The observed difference may partly be explained by the design of the previous studies: subjects including patients and attendees were recruited from an out-patient clinic of a private, fees-for-service health care facility which was accessed by patients from a relatively higher socio-economic strata and better health seeking behavior. On the other hand, people in community may have depression that is unrecognized or they may not have the resources to seek help. Furthermore, studies have reported that psychometric properties of GDS are weaker for cognitive impaired individuals [31] however; neither of the previous studies screened their participants for cognitive impairment which could have influenced their findings [22,23].

Previous literature has reported that elderly are more prone to depression than general population [15-20]. A systematic review showed that the burden of depression in general population in Pakistan is 34% [44]. The findings of our study substantiate the literature of higher prevalence of depression in elderly than the general population of Pakistan.

We calculated our sample size based on prevalence of 20% reported by the two previous studies [22,23], which might had resulted in underestimated sample size. However, our final sample size, after incorporating the proportion of family support, was much higher than what had been required at prevalence of 50%. Thus, the sample size was adequate to fulfill the objectives of the study.

With regard to family support, it is important to emphasize that Pakistan is a collectivistic society and family play a significant part in the life of elderly. Joint and extended family are traditionally well established and accepted norms. The elderly in Pakistan rarely live alone or fend for themselves after retirement. Most elderly not only expect to be looked after by their families but the families also consider this as their religious, social and moral obligation. Families provide economic as well as physical support to their retired parents [24-26]. In Pakistan, there is no state support for the elderly, who have to rely on their families for their social and economic needs [22,25].

One of the greatest needs of elderly is to have a companion and support of a life partner. On the contrary, the loss of spouse due to death, separation or divorce greatly reduces their capacity to face the challenges of life. The loss of a spouse can affect the psychosocial health as well as the survival of an individual at any stage of life. However, the elderly are particularly vulnerable as their dependency on a spouse increases with age [22,45,46]. A community based study conducted in Japan revealed that, separated or widowed elderly people had higher odds for depression as compared to their married counterparts. The results were similar for both elderly men and women [46].

In Pakistan, children (especially adult male children) become important sources of support for the older people [22,24,25]. Lack of children’s support can create a sense of insecurity and hence, increase their vulnerability to depression [22,27].

Several studies have reported the association of ‘perception of a supportive family’ and mental health [27,28]. A study conducted in a primary care setting in New York, USA revealed that insufficient family support was associated with increased psychological symptoms among elderly [27]. Another study in USA showed that perception of social support is associated with the overall functioning of depressed elderly [28]. This is in line with the findings of our study.

## Conclusion

Our study results estimated 40.6% of elderly in Karachi to be suffering from probable depression, with higher prevalence in women. Family support status was found to have a strong association with the mental health of elderly. There is need for further research in this important but neglected group of the population. There is also need for similar studies from other cities of Pakistan, so that a national picture of mental health of the elderly in Pakistan can be obtained. The findings of such studies can be used to inform policy and develop relevant preventative and intervention programs.

## Abbreviations

WHO: World Health Organization; GDS: Geriatric depression scale; LAMI: Low and middle income; DALYs: Disability adjusted life years; MMSE: Mini-mental state examination; FBS: Federal Bureau of Statistics; ERC-AKU: Ethical Review Committee-Aga Khan University; ADL: Activities of daily living; IADL: Instrumental Activities of daily living; AKU: Aga Khan University; SAS: Statistical analysis system; OR: Odd ratio.

## Competing interests

The authors declare that they have no competing interests.

## Authors’ contributions

MB contributed in conception, design, conduct, analysis and preparation of the manuscript. MSK provided valuable feedbacks in the conduct and implementation of study as well as in finalizing the manuscript. MMK as subject expert critically reviewed and contributed to manuscript writing. All authors read and approved the final manuscript.

## Pre-publication history

The pre-publication history for this paper can be accessed here:

http://www.biomedcentral.com/1471-244X/13/181/prepub

## Supplementary Material

Additional file 1 Questionnaire used for data collection. Click here for file
