# Supplementary material for: Depression in the elderly in Karachi, Pakistan: a cross sectional study
Source: BMC Psychiatry. 2013 Jul 3;13:181. doi: 10.1186/1471-244X-13-181 (PMC3704964; doi:10.1186/1471-244X-13-181)
Supplement: Additional file 1 — Questionnaire used for data collection. [file 1471-244X-13-181-S1.pdf]

QUESTIONNAIRE

Depression assessment in elderly population of Karachi, Pakistan: A cross sectional survey

Student of MSc. Epidemiology and Biostatistics
Class of 2009
Community Health Sciences, The Aga Khan University, Karachi
Phone #: 4864857

Questionnaire Number

☐ ☐

Name of Household Head: ___________________

Address: ___________________________________

Name of Locality / Area: ____________________

Cluster Number: __________________________

Household Number: ________________________

INTERVIWER'S VISITS				
	**1**	**2**	**3**	**Final Visit**
**Date**	______________	_______________	_______________	**Day__________**
**Interviewer Name**	______________	_______________	_______________	**Month_______** **Year_________** **Inte #_________**
**Result**	______________	_______________	_______________	**Result_______**
**Next Visit Date:** **Time:**	______________ ______________	_______________ _____________	_______________ _______________	**Total Number of Visits_______**

Result Codes:

1. Completed
2. No household member at home or no competent respondent at home at time of visit
3. Entire household absent for extended period of time
4. Postponed
5. Refused
6. House vacant or address not a house
7. House destroyed
8. House not found
9. Others-----------

FIELD SUPERVISOR	FIELD MANAGER	PRINCIAL INVESTIGATOR	DATA ENTRY
NAME____________ ____________________ DATE____________	NAME____________ ____________________ DATE____________	NAME____________ ____________________ DATE____________	NAME____________ ____________________ DATE____________

<table>
<tr><th colspan="5">HOUSEHOLD QUESTIONNAIRE</th></tr>
<tr><th>S.No.</th><th>Questions</th><th>Code</th><th>Skip</th><th>Response</th></tr>
<tr><td>A1</td><td>How many persons usually live in this household?</td><td>88 = Don’t know
99 = No response</td><td></td><td>-----------------</td></tr>
<tr><td>A2</td><td>What is the type of family you have? Is it joint or nuclear or other than these mentioned?</td><td>1 = Joint,
2 = Nuclear
3 = Alone
Others (specify)</td><td></td><td>-----------------</td></tr>
<tr><td>A3</td><td>Is it your own or rented accommodation?</td><td>1 = Family owned,
2 = Rented,
3 = Owned by other relative,
4 = Official residence,
Others (specify)</td><td></td><td>--------------------</td></tr>
<tr><td>A4</td><td>What is your Religion?</td><td>1=Muslim,
2=Hindu,
3=Christian,
4=Parsi,
Other (specify)</td><td></td><td>------------------</td></tr>
</table>

HOUSEHOLD SCHEDULE<br>We would like to obtain information about the people who usually live in this household								
**Line No.**	**Usual Residents**	**Relationship with the head of household (see codes below)**	**Sex**	**Age**	**Education (See codes below)**	**Marital Status**	**Occupation (See codes below)**	**Ask if not working (retired) For past how many years is he/she not working?**
	Please give me the names of persons who usually live in the household	What is the relationship to the head of household?	1=Male.<br>2=Female	In completed years		1=Married<br>2=Widowed<br>3=Separated<br>4=Divorced<br>5=Never married	If working skip to C1	
**B1**	**B2**	**B3**	**B4**	**B5**	**B6**	**B7**	**B8**	**B9**
i								
ii								
iii								
iv								
v								
vi								
vii								
viii								

Codes Relationship to Head of Household:
1=Head, 2=Wife or husband, 3=Son or daughter,
4=Son in law or daughter in law, 5=Grandchild,
6=Parent, 7=Parent in law, 8=Brother or sister,
9=Other relative, 10=-Adopted/step child,
11=Not related, Others specify,
88=Don't know, 99= No response

Codes for Education:
1=Illiterate, 2=Literate (no formal schooling),
3=Upto 5th, 4=Upto Matric, 5=11-14 class,
6=MA/MSc./MBBS/MBA or above,
Others specify,88= Don't know, 99=No response.

Codes for Occupation:

Working:
1=Professional/Executive, 2=Manager/Supervisor,
3=Office/clerk work, 4=Sales workers, 5=Skilled factory worker,
6=Skilled non factory worker, 7=Labourer, 8=Household helpers (drivers, cook, chawkidaar), 9=Commercial vehicle operators,
10=Self employed, 11=Small venders, 12=Agriculturist,
13=Business, Others specify, 88= Don't know, 99=No response

Non working:
15=retired and gets pension, 16= retired and doesn't get pension,
17=too old/ill to work, 18=disabled, 19=Unemployed,
20=housewife, 77=Others specify,
88= Don't know, 99=No response.

HOUSING CHARACTERISTICS & LIVING STANDARDS				
**S.No**	**Questions**	**Coding**	**Skip**	**Response**
**C1**	What is the main source of drinking water in this household?	1 = Independent tap<br>2 = Public tap<br>3 = Own hand pump<br>4 = Public hand pump<br>5 = Public well<br>6 = Tanker<br>7 = Mineral water bottle<br>88 = Don't know<br>99 = No response<br>Others specify		------------------
**C2**	How many rooms are in this house? (excluding verandah and kitchen)	88 = Don't know<br>99 = No response		--------------------
**C3**	What toilet facility is available to you?	1=Flush toilet<br>2=Traditional dry toilet<br>3=Public latrine<br>4=No facility on Premises<br>88=Don't know<br>99=No response<br>Others specify		--------------------
**C4**	Does your household have any of the following for self use:<br>A. Air conditioner	1 = Yes<br>2 = No<br><br>If yes, how many		________________<br><br>________________
	B. Personal Computer	1 = Yes<br>2 = No<br><br>If yes, how many		________________<br><br>________________
	C. Internet	1 = Yes<br>2 = No		________________
	D. Car/Van/Suzuki for commercial use	1 = Yes<br>2 = No<br><br>If yes, how many		________________<br><br>________________

	E. Car for personal use	1 = Yes<br>2 = No<br><br>If yes, how many		______________<br><br>______________
	F. Motor cycle/scooter	1 = Yes<br>2 = No<br><br>If yes, how many		______________<br><br>______________
	G. Video player/VCD	1 = Yes<br>2 = No<br><br>If yes, how many		______________<br><br>______________
	H. Television	1 = Yes<br>2 = No<br><br>If yes, how many		______________<br><br>______________
	I. TV cable	1 = Yes<br>2 = No		______________
	J. Refrigerator	1 = Yes<br>2 = No<br><br>If yes, how many		______________<br><br>______________
	K. Washing Machine	1 = Yes<br>2 = No<br><br>If yes, how many		______________<br><br>______________
	L. Telephone	1 = Yes<br>2 = No<br><br>If yes, how many		______________<br><br>______________
	P. Electric Fan	1 = Yes		______________

		2 = No<br><br>If yes, how many		______________
	N. Electric Iron	1 = Yes<br>2 = No<br><br>If yes, how many		______________<br><br>______________
**C5**	What type of fuel does your household mainly use for cooking?	1=Electricity<br>2=Sui gas<br>3=Burshane<br>4=Kerosene<br>5=Coal<br>6=Charcoal<br>7=Firewood<br>8=Cow dung<br>9=Food from hotel<br>Others(specify)		------------------
**C6**	How is your house constructed?	1=Cemented<br>2=Wall cemented but roof uncemented<br>3=Uncemented<br>4=Wooden house<br>**5=**Slum		--------------------
**C7**	What is the temporary source of light in the absence of electricity in your house?	1=Generator<br>2=UPS<br>3=Emergency light<br>4=Gas light<br>5=Lantern<br>6=Candle<br>7=Nothing<br>Others specify		-------------------

PERSONAL INFORMATION

S.No	Questions	Coding	Skip	Response
**D1**	What is your age in years? (Give them hint like how older were you at the time of independence)			Confirm with B5 as well<br>----------------
**D2**	What is your birth place?	1=Karachi<br>2=Interior Sindh<br>3=Punjab<br>4=NWFP		----------------

		5=Balochistan<br>6=Kashmir<br>7=Tribal areas<br>8=Northern areas<br>9=India<br>10=Abroad (other than India)		
**D3**	What is your Education level?	1=Illiterate<br>2=Literate (no formal schooling)<br>3=Upto 5th<br>4=Upto Matric<br>5=11-14 class<br>6=MA/MSc./MBBS/ MBA or above<br>88= don't know<br>99=no response<br>Others specify		-----------------
**D4**	How many live births you/ your wife got?		If none than skip to D6	-----------------
**D5**	How many children are still alive?			------------------
**D6**	Do you think children are security for future?	1=Yes<br>2=No<br>88=Don't know		-<br>----------------
**D7**	With whom you live whether nuclear or extended?	1=Extended (with married son/sons<br>2=Extended (with married daughter/daughters<br>3= Extended (with other relatives-not own children)<br>4= Extended (with own children and other relatives)<br>5= Extended (with unmarried children)<br>6=With spouse<br>7=Alone<br>Other specify		----------------
**D8**	Do you have separate room	1 = Yes		------------------

	for sleeping purposes just for you and/or your spouse (if present)	2 = No<br>99 = No response		
**D9**	What is your mother tongue?	1=Urdu<br>2=Sindhi<br>3=Punjabi<br>4=Balochi<br>5=Pashto<br>6=Hindko<br>7=Saarki<br>8=Gujrati<br>9=Memon<br>10=Katchi<br>11=Gorshi<br>12=Marwari<br>13=Rajhistani<br>Others (specify)		------------------
**D10**	Do you work to earn money?	1=Yes<br>2=No<br>88=Don't know<br>99=No response	If no, then skip to D12	------------------
**D11**	If working to earn, what is your total monthly income from all sources?	88 = Don't know<br>99 =No response	Skip to D13	------------------
**D12**	If not working to earn, what is your total monthly income from all sources? (e.g.: pension, from children, home rentals, business income etc.)	88 = Don't know<br>99 =No response		------------------
**D13**	Have you ever been married?	1=Yes,<br>2=No	If no, then skip to D37	------------------
**D14**	How many years you have been or were married?	88 = Don't know<br>99 =No response		------------------
**D15**	What is your current Marital Status?	1=Married<br>2=Widowed<br>3=Separated<br>4=Divorced	If answer other than married skip to D17	------------------

**D16**	If married how long you have been married with your present spouse? (years)	88 = Don't know<br>99 =No response	Skip to D18	------------------
**D17**	If widowed or separated or divorced, how long you have been widowed/widower or separated or divorced? (years)	88 = Don't know<br>99 =No response		------------------
**D18**	How many adult (above 18 years) children do you have who are alive?		If none, then skip to D37	------------------
**D19**	How many are sons that are above 18 years?			------------------
**D20**	How many are daughters that are above 18 years?			------------------
**D21**	How many of your adult (above 18 years) children are living with you?			------------------
**D22**	Do you have any of your adult (above 18 years) children who are living in this moholla but not with you?	1 = Yes<br>2 = No	If no or skip to D24	------------------
**D23**	How many of your adult (above 18 years) children are living in this Moholla but not with you?			------------------
**D24**	Do you have any of your adult (above 18 years) children who are living else where in Karachi but not with you?	1 = Yes<br>2 = No	If no skip to D26	------------------
**D25**	How many of your adult (above 18 years) children are living else where in Karachi but not with you?			------------------
**D26**	Do you have any of your adult (above 18 years) children who are living else where in Pakistan?	1 = Yes<br>2 = No	If no skip to D28	------------------
**D27**	How many of your adult (above 18 years) children are living else where in Pakistan?			------------------
**D28**	Do you have any of your adult (above 18 years) children who are living abroad?	1 = Yes<br>2 = No	If no skip to D35	------------------
**D29**	How many of your adult			

	(above 18 years) children are living abroad?			------------------
**D30**	Do you miss your adult (above 18 years) children who are living outside Pakistan?	1=Yes<br>2=No	If No skip to D32	------------------
**D31**	If yes, how much do you miss them?	1=Very often<br>2=Whenever I am lonely<br>3=Rarely<br>88=Don't know/can't say		------------------
**D32**	Do you get telephone calls from them or you call them?	1 = I neither call them nor they do<br>2 = I call them<br>3 = They call me<br>4 = We both call each other	If 2 or 4 then ask D33, If 3 skip to D34, If 1 skip to D35	------------------
**D33**	How often do you call them?	1=Daily<br>2= Weekly<br>3=Twice a week<br>4=Once in a month<br>5=Once in 3 months<br>6=Once in 6 months<br>7=Once in a year<br>8=Not done<br>Other specify		------------------
**D34**	How often do they call you?	1=Daily<br>2= Weekly<br>3=Twice a week<br>4=Once in a month<br>5=Once in 3 months<br>6=Once in 6 months<br>7=Once in a year<br>8=Not done<br>Other specify		------------------
**D35**	How often your children living inside moholla our elsewhere in Karachi pay visit to you?	1=Daily<br>2= Weekly<br>3=Twice a week<br>4=Once in a month<br>5=Once in 3 months<br>6=Once in 6 months<br>7=Once in a year<br>8=Not done<br>Other specify		Moholla<br>________________<br>Karachi<br>________________
**D36**	How often do you pay visit to	1=Daily		

	your children living inside moholla our elsewhere in Karachi?	2= Weekly<br>3=Twice a week<br>4=Once in a month<br>5=Once in 3 months<br>6=Once in 6 months<br>7=Once in a year<br>8=Not done<br>Other specify		Moholla<br>______________<br>Karachi<br>______________
**D37**	How would you rate your health at present time?	1=Very good<br>2=Fairly good<br>3=Good<br>4=Poor<br>88=Can't say/don't know<br>99=No response		-----------------
**D38**	How your functional Health is today compared to what it was last year?	1=Better<br>2=About the same<br>3=Worse<br>88=Can't say/don't know<br>99=No response		-----------------

**ASSESSMENT OF PHYSICAL ACTIVITY**				
**Upper Extremity Domain**				
**S.No**	**Questions**	**Coding**	**Skip**	**Response**
E1	By yourself do you have any difficulty raising your arms up over your head? If yes since how long? (mention in days, weeks, months)	1= Yes<br>2= No<br>88= Don't Know<br>99= No Response		---------------
E2	By yourself do you have any difficulty using your fingers to grasp or handle? If yes since how long? (mention in days, weeks, months)	1= Yes<br>2= No<br>88= Don't Know<br>99= No Response		---------------
E3	By yourself do you have any difficulty lifting or carrying something as heavy as 5 kilogrammes? If yes since how long? (mention in days, weeks, months)	1= Yes<br>2= No<br>88= Don't Know<br>99= No Response		---------------
**Lower Extremity Domain**				
**S.No**	**Questions**	**Coding**	**Skip**	**Response**
E4	By yourself do you have any difficulty walking for about 200-250 yards? If yes since how long? (mention in days, weeks, months)	1= Yes<br>2= No<br>88= Don't Know<br>99= No Response		---------------
E5	By yourself do you have any difficulty walking up to 10-15 steps without resting? If yes since how long? (mention	1= Yes<br>2= No<br>88= Don't Know		---------------

	in days, weeks, months)	99= No Response		
E6	By yourself do you have any difficulty getting in and out of bed or chairs/table? If yes since how long? (mention in days, weeks, months)	1= Yes<br>2= No<br>88= Don't Know<br>99= No Response		---------------
	**ADL Domain**			
**S.No**	**Questions**	**Coding**	**Skip**	**Response**
E7	By yourself that is without help from others or special equipment do you have any difficulty in bathing or showering? If yes since how long? (mention in days, weeks, months)	1= Yes<br>2= No<br>88= Don't Know<br>99= No Response		---------------
E8	By yourself that is without help from others or special equipment do you have any difficulty in dressing? If yes since how long? (mention in days, weeks, months)	1= Yes<br>2= No<br>88= Don't Know<br>99= No Response		---------------
E9	By yourself that is without help from others or special equipment do you have any difficulty in eating, for example: holding folk or spoon, cutting your food or drinking from a glass? If yes since how long? (mention in days, weeks, months)	1= Yes<br>2= No<br>88= Don't Know<br>99= No Response		---------------
E10	By yourself that is without help from others or special equipment do you have any difficulty using the toilet, including getting up from the toilet seat or commode? If yes since how long? (mention in days, weeks, months)	1= Yes<br>2= No<br>88= Don't Know<br>99= No Response		---------------
	**IADL Domain**			
**S.No**	**Questions**	**Coding**	**Skip**	**Response**
E11	Because of your health status do you have difficulty in using telephone? If yes since how long? (mention in days, weeks, months)	1= Yes<br>2= No<br>88= Don't Know<br>99= No Response		---------------
E12	Because of your health status do you have difficulty doing light house work like washing dishes, light cleaning etc? If yes since how long? (mention in days, weeks, months)	1= Yes<br>2= No<br>88= Don't Know<br>99= No Response		---------------
E13	Because of your health status do you have difficulty doing heavy household work like washing clothes or cleaning floor etc? If yes since how long? (mention in days, weeks, months)	1= Yes<br>2= No<br>88= Don't Know<br>99= No Response		---------------
E14	Do you have difficulty preparing your own meal? If yes since how long? (mention in days, weeks, months)	1= Yes<br>2= No<br>88= Don't Know		---------------

		99= No Response		
E15	Will (Do) you have difficulty if need to go for (going for) shopping for your personal items including vegetables or medicine etc? If yes since how long? (mention in days, weeks, months)	1= Yes<br>2= No<br>88= Don't Know<br>99= No Response		---------------

Other Activities

S.No	Questions	Coding	Skip	Response
E16	Do you go to walk for pleasure or exercise?	1= Yes<br>2= No	If No, skip to E20	
E17	During an average week, how many hours do you spend walking for pleasure or exercise?			______ Hrs
E18	Since how long you have been going for walking?	88=Don't know		______ Months<br>______ Years
E19	When you walk for pleasure or exercise what usually happens to the rate or depth of your breathing?	1= No change<br>2= Slight increase<br>3=Moderate increase<br>4=Severe increase	Skip to E21	______
E20	Since how long you have not been going for walking?	88=Don't know		______ Months<br>______ Years
E21	Do you lift or carry objects that are as heavy as 5 kg? (do not include lifting or carrying objects at work place)	1= Yes<br>2= No	If no then skip to E23	______
E22	During an average week, how much time do you spend lifting or carrying objects as heavy as 5 kg? (do not include time spent lifting or carrying objects at work place)			______ Min<br>______ Hrs
E23	Which of the following four activity classes best describes your present activity outside? Please consider to and from work and other physical effort during your leisure time?	1= No weekly physical activity<br>2=Only light physical activity in most weeks		______

		3=Vigorous physical activity at least 20 min 1s or twice a week (vigorous activity causes shortness of breadth, a rapid heart rate & sweating)<br>4=Vigorous physical activity for at least 20 minutes 3 or > times per week		
E24	Do you go to meet any of your relative or friend?	1= Yes<br>2= No	If no skip to E26	
E25	During an average week, how much time do you spend meeting your friends or relatives?			______ Min<br>______ Hrs
E26	Do you spend time in performing various activities like gardening etc?	1= Yes<br>2= No	If no skip to E228	______
E27	During an average week, how much time do you spend performing various activities like gardening etc?			______ Min<br>______ Hrs
E28	Do you go outside home for any other purpose example to purchase items etc?	1 = Yes<br>2 = No	If no skip to E30	______
E29	During an average week, how much time do you spend going out for any other purpose example to purchase items etc?			______ Min<br>______ Hrs

Working or Employment Activities

S.No	Questions	Coding	Skip	Response
E30	Are you employed?	1=Yes<br>2=No	If yes skip to E32	______

E31	If not working, then when you left working (in months or years)?		Skip to F1	________ Months<br><br>________ Years
E32	How many hours do you work during a typical day?	88=Can't say/don't know<br>99=No response		________ Hrs
E33	How many days do you work during a typical week?	88=Can't say/don't know<br>99=No response		________ Days
E34	How much time during work do you spend standing or sitting?			**Standing**<br>________ Min<br>________ Hrs<br>**Sitting**<br>________ Min<br>________ Hrs
E35	How much time during work do you spend walking? (don't include time of from and to work)			________ Min<br>________ Hrs
E36	How much time during work do you spend lifting weight up to 5kg or doing exercises like this?			________ Min<br>________ Hrs

ASSESSMENT OF DEPRESSION (GDS)				
**S.No**	**Questions**	**Coding**	**Skip**	**Response**
F1	Are you basically satisfied with your life?	0= No<br>1=Yes		
F2	Have you dropped many of your activities and interests?	0= No<br>1=Yes		
F3	Do you feel that your life is empty?	0= No<br>1=Yes		
F4	Do you often get bored?	0= No<br>1=Yes		
F5	Are you in good spirits most of the time?	0= No<br>1=Yes		
F6	Are you afraid that something bad is going to happen to you?	0= No<br>1=Yes		
F7	Do you feel happy most of the time?	0= No<br>1=Yes		
F8	Do you often feel helpless?	0= No<br>1=Yes		
F9	Do you prefer to stay at home, rather than going out and doing new things?	0= No<br>1=Yes		
F10	Do you feel that you have more problems with memory than most?	0= No<br>1=Yes		
F11	Do you think it is wonderful to be alive now?	0= No<br>1=Yes		
F12	Do you feel pretty worthless the way you are now?	0= No<br>1=Yes		
F13	Do you feel full of energy?	0= No<br>1=Yes		
F14	Do you feel that your situation is hopeless?	0= No<br>1=Yes		
F15	Do you think that most people are better off than you are?	0= No<br>1=Yes		
